# Antiviral Activity of Isoimperatorin Against Influenza A Virus *in vitro* and its Inhibition of Neuraminidase

**DOI:** 10.3389/fphar.2021.657826

**Published:** 2021-04-13

**Authors:** Yanni Lai, Tiantian Han, Shaofeng Zhan, Yong Jiang, Xiaohong Liu, Geng Li

**Affiliations:** ^1^ Guangzhou University of Chinese Medicine, Guangzhou, China; ^2^ The First Affiliated Hospital of Guangzhou University of Chinese Medicine, Guangzhou, China; ^3^ Shenzhen Hospital of Integrated Traditional Chinese and Western Medicine, Shenzhen, China; ^4^ Laboratory Animal Center, Guangzhou University of Chinese Medicine, Guangzhou, China

**Keywords:** isoimperatorin 1, antiviral 2, influenza 3, neuraminidase 4, docking 5

## Abstract

Influenza A virus (IAV) poses a severe threat to human health and is a major public health problem worldwide. As global anti-influenza virus drug resistance has increased significantly, there is an urgent need to develop new antiviral drugs, especially drugs from natural products. Isoimperatorin, an active natural furanocoumarin, exhibits a broad range of pharmacologic activities including anticoagulant, analgesic, anti-inflammatory, antibacterial, anti-tumor, and other pharmacological effects, so it has attracted more and more attention. In this study, the antiviral and mechanistic effects of isoimperatorin on influenza A virus *in vitro* were studied. Isoimperatorin illustrated a broad-spectrum antiviral effect, especially against the A/FM/1/47 (H1N1), A/WSN/33 (H1N1, S31N, amantadine resistant), A/Puerto Rico/8/34 (H1N1), and A/Chicken/Guangdong/1996 (H9N2) virus strains. The experimental results of different administration modes showed that isoimperatorin had the best antiviral activity under the treatment mode. Further time-of-addition experiment results indicated that when isoimperatorin was added at the later stage of the virus replication cycle (6–8 h, 8–10 h), it exhibited an effective antiviral effect, and the virus yield was reduced by 81.4 and 84.6%, respectively. In addition, isoimperatorin had no effect on the expression of the three viral RNAs (mRNA, vRNA, and cRNA). Both the neuraminidase (NA) inhibition assay and CETSA demonstrated that isoimperatorin exerts an inhibitory effect on NA-mediated progeny virus release. The molecular docking experiment simulated the direct interaction between isoimperatorin and NA protein amino acid residues. In summary, isoimperatorin can be used as a potential agent for the prevention and treatment of influenza A virus.

## Introduction

Influenza virus, a single-stranded negative-strand RNA virus of *Orthomyxoviridae*, is an important respiratory pathogen that has a significant impact on global health. Due to the high mutation rate of virus genes, effective virus transmission, rapid emergence of drug resistance and the limited effectiveness of currently available therapies, the spread of the virus can easily cause a pandemic. Since the global influenza pandemic in 1918, the outbreak caused by the influenza virus, this virus has caused 50 million deaths in this century (Jester et al., 2019). Influenza A viruses are divided into many subtypes according to H and N antigens. Among them, H1N1, H2N2, and H3N2 mainly infect humans. Currently, there are three types of effective anti-influenza drugs approved by the FDA. These drugs are M2 channel blockers (rimantadine and amantadine) (De Clercq, 2006), an RNA polymerase inhibitor (ribavirin) and neuraminidase inhibitors (oseltamivir and zanamivir) (Doshi and Jefferson, 2016). However, with the emergence of viral resistance and the high prices of drugs, the discovery and development of new antiviral drugs has become more urgent.

The replication cycle of influenza A virus (IAV) mainly includes the steps of adsorption, penetration, uncoating, and biosynthesis. When IAV invades host cells, the hemagglutinin (HA) located on the surface of the virus envelope first binds to sialic acid residues expressed by airway or alveolar epithelial cells, triggering the endocytosis of virus particles. Acidification of the endosome results in fusion of viral HA and the endosomal membrane and activation of the M2 ion channel, allowing protons to enter the virus core to dissociate the ribonucleoprotein complex and then transport it into the nucleus, where the virus replicates (Huang et al., 2001). After the virus has replicated, it assembles, buds, and breaks on the lipid rafts on the cell membrane (Hutchinson and Fodor, 2013). After breaking, the HA in the newly formed virosome binds to the sialic acid receptor on the cell surface. When these connections are cleaved by NA, progeny viruses are released to infect other cells or leave the individual through respiratory secretions (Dou et al., 2018). Therefore, NA has been the most important target for the development of novel anti-influenza drugs.

Isoimperatorin, an active natural furanocoumarin, is a component of the traditional Chinese herbal medicines *Angelica dahurica*, *Rhizoma et Radix Notopterygii* and *Radix Peucedani*. In addition, isoimperatorin also exists in lemon oil and lime oil. Isoimperatorin exhibits a broad range of pharmacological activities, including analgesic, anti-inflammatory, antibacterial, antitumour, diastolic vasoactivity, liver protection and other pharmacological effects, so it has attracted increasing attention (Nie et al., 2009; Guo et al., 2014; Tong et al., 2017; Wijerathne et al., 2017). A recent study has shown that isoimperatorin may have antiviral effects on herpes simplex virus-1 and coxsackievirus B3 (Rajtar et al., 2017). Furthermore, it may could anti-H1N1 and H9N2 influenza virus *in vitro* (Lee et al., 2020). However, its antiviral mechanism of action is still unclear.

In the present study, we investigated the antiviral effect and mechanism of action of isoimperatorin against influenza infection. We first identified the inhibitory effects of isoimperatorin against the influenza A/FM/1/47 (H1N1), A/WSN/33 (H1N1, S31N, amantadine resistant), A/Puerto Rico/8/34 (H1N1), A/Chicken/Guangdong/1996 (H9N2), A/Hong Kong/498/97 (H3N2) and B/Lee/1940 (IVB) strains. The effects of isoimperatorin on H1N1 replication and neuraminidase activity were assayed using A/PR/8/34 (H1N1), and our results indicate that isoimperatorin can be used as a neuraminidase inhibitor to manage influenza virus infection.

## Materials and Methods

### Cells and Virus

A/FM/1/47 (H1N1), A/WSN/33 (H1N1, S31N, amantadine resistant), A/Puerto Rico/8/34 (H1N1), A/Chicken/Guangdong/1996 (H9N2), A/Hong Kong/498/97 (H3N2) and influenza B/Lee/1940 (IVB) were propagated in 9 day-old specific pathogen-free (SPF) embryonated hen eggs. The titre of viruses was detected by plaque assays. The viruses were stored at −80°C in the Laboratory Animal Center of Guangzhou University of Chinese Medicine (Guangzhou, China).

Madin-Darby canine kidney (MDCK) cells were maintained in Dulbecco's modified Eagle's medium (DMEM) supplemented with 10% foetal bovine serum, 100 U/ml penicillin, and 100 U/ml streptomycin at 37 C under 5% CO_2_. In anti-influenza assays, DMEM containing 2 μg/ml tosyl phenylalanyl chloromethyl ketone (TPCK)-treated trypsin and 1% bovine serum albumin was used.

### Compounds

Isoimperatorin (purity ≥98%) was purchased from Baoji Herbest Bio, dissolved in dimethyl sulfoxide (DMSO) as a storage solution, and then diluted with serum-free DMEM into working solutions of different concentrations. The final DMSO concentration was 0.1%. Ribavirin (purity ≥98%) and oseltamivir (purity ≥ 98%) were purchased from Shanghai Yuanye Biotechnology Co., Ltd. and diluted with serum-free DMEM into working solutions of different concentrations.

### Cytotoxicity Assay

The cytotoxic activities of isoimperatorin on MDCK cells were assessed by the WST-8 assay using a Cell Counting Kit-8 (Dojindo, Kumamoto, Japan) (Zhang et al., 2019). Cells (1 × 10^5^ cells/well) were seeded into 96-well plates and incubated overnight. The cells were cultured with various concentrations of isoimperatorin (0, 20, 40, 60, 100, 120, 140, 160, and 200 μM) at 37 C under 5% CO_2_ for 48 h. After 48 h, the culture medium was removed, and 10% WST-8 reagent in a volume of 100 µl was added to each well and incubated at 37 C for 30 min. We measured absorbance at a wavelength of 450 nm with a multimode microplate reader (PerkinElmer). The cell viability was expressed as the percentage compared with the DMSO control group designated as 0.1%. Cell viability rate (%) = (mean optical density of test/mean optical density of cell controls) × 100.

### Cytopathic Effect (CPE) Inhibition Assay

The CPE experiment method refers to the previous experiment (Zhou et al., 2017), and the details are as follows: MDCK cells (2 × 10^4^ cells/well, 100 μl) were seeded into each well of 96-well plastic plates and cultured at 37 C under 5% CO_2_ for 24 h. For the anti-influenza activity assay, MDCK cells were inoculated with a total of 100 TCID_50_ of the influenza virus infective titre at 37 C for 2 h. Then, cells were washed and cultured for 48 h at 37 C under 5% CO_2_ in the presence of 100 μl of isoimperatorin (0.09–100 μM) in DMEM supplemented with 2 μg/ml TPCK-trypsin (Sigma, Unites States). After 48 h, CPE in virus-infected cells was observed by light microscopy. Cell viability was measured using Cell Counting Kit-8. The EC_50_ values of the test compounds were determined by fitting the curve of percent CPE vs. the compound concentrations using GraphPad Prism 5.

The inhibition rate of the test compounds was calculated using the following equation (Yu et al., 2016): inhibition rate (%) = [(mean optical density of test–mean optical density of virus controls)/(mean optical density of cell controls–mean optical density of virus controls)] × 100. Each value is an average from three independent experiments. The selectivity index (SI) was calculated from the ratio of median toxic concentration (TC_50_)/concentration for 50% of the maximal effect (EC_50_).

### Plaque Reduction Assay

The experimental method of plaque reduction refers to a previous description (Furuta et al., 2002), as follows: MDCK cells (6 × 10^5^ cells/well, 2 ml) were seeded into each well of 12-well plates and cultured at 37 C under 5% CO_2_ for 24 h. Then, the cells were washed with PBS and infected with the supernatants collected from virus-infected cells treated with the test compounds for 2 h. The virus inoculums were removed, and the cells were washed with PBS three times. Next, the cell monolayers were overlaid with agar overlay medium (DMEM supplemented with 1% low melting point agarose and 2.5 μg/ml TPCK-treated trypsin) and incubated at 37 C for 3–4 days. The cell monolayers were fixed with 4% paraformaldehyde for 1 h. The agarose overlays were then removed, and the cell monolayers were stained with 2% (w/v) crystal violet.

### The Methods of Three Modes of Medication

**FIGURE 1:**
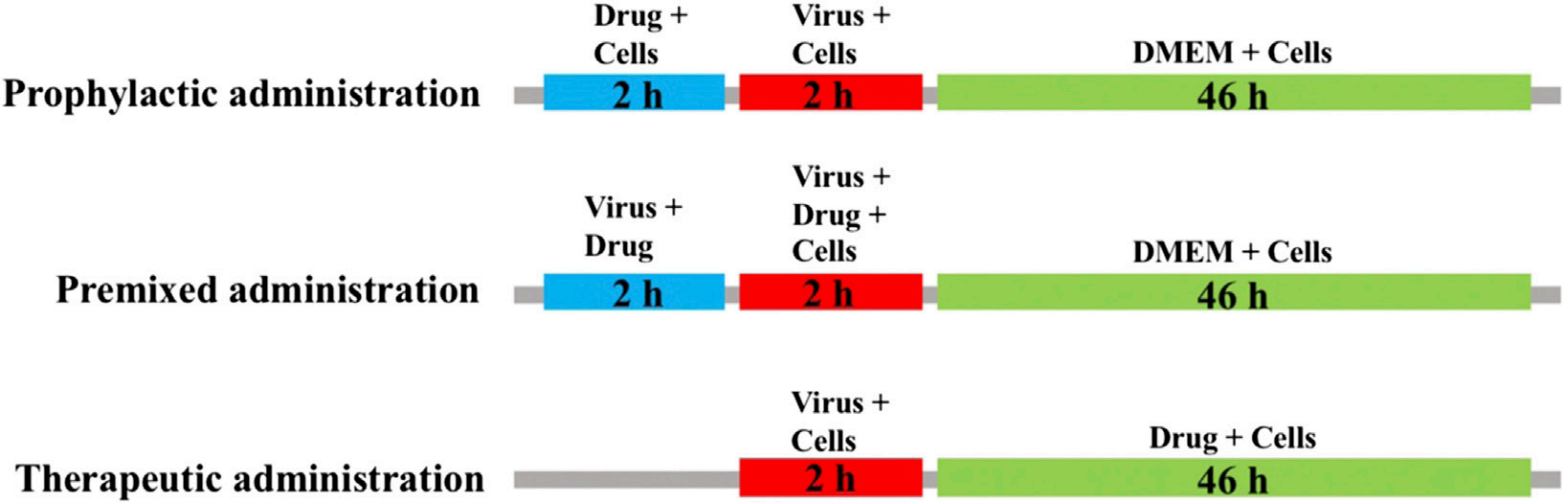
Sketch of three medication modes. Prophylactic administration: drugs added to the cell culture 2 h before the infectious virus was added to MDCK cells. Premixed administration: drugs and virus were mixed and incubated at 4 C for 2 h, and then the mixture was added to the cell culture and incubated at 4 C for 2 h. Therapeutic administration: drugs were added to MDCK cells after infection by the virus.

MDCK cells were seeded into 96-well plates and cultured at 37 C under 5% CO2 for 24 h. The blank control group, virus control group and sample group were set up. After 2 h of virus infection, cells were washed 3 times with PBS and then switched to serum-free medium containing 2 μg/ml TPCK-trypsin (Sigma, United States) and different concentrations of isoimperatorin solution. The administration mode is shown in Figure 1, including preventive administration, premixed administration and therapeutic administration. After 48 h, a CCK-8 kit was used to detect absorbance under a multimode microplate reader (450 nm).

### Time of Addition Assay

A time-of-addition experiment was conducted as previously described (Zhang et al., 2019). The confluent monolayers of MDCK cells in 12-well plates were inoculated with 100 TCID_50_ of virus at 37 C for 2 h (time 0–2 h). Then, the monolayers were washed three times with PBS three times and incubated with DMEM containing 2.5 μg/ml TPCK-treated trypsin at 37 C. Test medium containing isoimperatorin (12.5, 25, and 50 μM) was added at seven points (−2–0, 0–2, 2–4, 4–6, 6–8, 8–10, and 10–24 h). After each internal incubation, the cells were washed with PBS three times and incubated with fresh medium (containing 2.5 μg/ml TPCK-treated trypsin). At 48 h postinfection, the monolayers were collected and frozen at −80°C for RT-PCR analysis.

### Quantitative Real-Time RT-PCR Analysis

Total RNA from cells was extracted using the Ultrapure RNA kit (CoWin Biotech, Beijing, China), and cDNA was then synthesized from the total RNA using the M-MLV Reverse Transcriptase kit (Promega, Madison, WI, United States) with random primers and oligo (dT) primers. For the three viral RNA species, reverse transcription (RT) was conducted using NP specific oligonucleotides for vRNA, cRNA, and mRNA. The PCR system was performed with 25 µl reaction buffer (17 µl of RNA template, 5 µl of M-MLV 5× reaction buffer, 50 pmol of the primers, 0.1 mM dNTPs, 25 units of ribonuclease inhibitor, and 200 units of M-MLV RT polymerase). The cycle conditions of qPCR were 1 cycle at 95 C for 3 min, followed by 39 cycles of 95 C for 10 s, 60 C for 10 s, 70 C for 20 s and 1 cycle at 95 C for 10 s.

The specific primers were described as followed:

qPCR_Fw-NPmRNA: 5′-CAT​CTT​TCT​GGC​ACG​GTC​TG-3′

qPCR_Rv-NPmRNA: 5′-GGC​TAC​TGC​AGG​TCC​ATA​CA-3′

qPCR_Fw-GAPDHmRNA: 5′-AAC​ATC​ATC​CCT​GCT​TCC​AC-3′

qPCR_Rv-GAPDHmRNA: 5′-GAC​CAC​CTG​GTC​CTC​AGT​GT-3′

RT NPvRNA: 5′-GGC​CGT​CAT​GGT​GGC​GAA​TGA​ATG​GAC​GAA​AAA​CAA​GAA​TTG​C-3′

RT NPcRNA: 5′-GCT​AGC​TTC​AGC​TAG​GCA​TCA​GTA​GAA​ACA​AGG​GTA​TTT​TTC​TTT-3′

RT NPmRNA: 5′-CCA​GAT​CGT​TCG​AGT​CGT​TTT​TTT​TTT​TTT​TTT​TCT​TTA​ATT​GTC-3′

qPCR_Fw-NPvRNA: 5′-CTC​AAT​ATG​AGT​GCA​GAC​CGT​GCT-3′

qPCR_Fw-NPcRNA: 5′-CGA​TCG​TGC​CTT​CCT​TTG-3′

qPCR_Fw-NPmRNA: 5′-CGA​TCG​TGC​CTT​CCT​TTG-3′

Tag_Rv-NPvRNA: 5′-GGC​CGT​CAT​GGT​GGC​GAA​T-3′

Tag_Rv-NPCRNA: 5′-GCT​AGC​TTC​AGC​TAG​GCA​TC-3′

Tag_Rv-NPmRNA: 5′-CCA​GAT​CGT​TCG​AGT​CGT-3′

### Western Blot Analysis

MDCK cells were collected and lysed in CO-IP lysis buffer for 40 min. Then, the cells were centrifuged at 12,000 × *g* for 10 min at 4 C. Proteins were analysed by SDS-PAGE and then transferred to PVDF membranes (Bio-Rad, California, United States). Five percent milk solution was used to block the membrane at room temperature for 1 h, and then the membrane was incubated with the primary antibody overnight at 4 C. The protein signal was visualized using ECL Western blotting reagent (Bio-Rad, California, Unites States). The NP antibody and NA antibody used to detect influenza virus and the antibody to detect GAPDH were all purchased from GENE Tex (Southern California, Unites States).

### NA Inhibition Assay

A neuraminidase assay kit (Beyotime) was used to analyse NA activity. The detection steps were carried out in accordance with the manufacturer's instructions. Briefly, a positive control, a negative control and a sample group were set up separately. Ten microlitres of isoimperatorin of different concentrations was added to 70 μl of detection buffer, 10 μl of water and 10 μl of NA fluorescent substrate were added, and the mixture was incubated at 37 C for 30 min. A multifunctional microplate reader (PerkinElmer) was used to measure the fluorescence intensity by excitation at 322 nm and emission at 450 nm.

### Cellular Thermal Shift Assay (CETSA)

As mentioned in the previous literature (Martinez Molina et al., 2013), CETSA measurement was performed with some modifications. In brief, MDCK cells were collected and washed with PBS and then resuspended in detergent-free buffer supplemented with a mixture of protease inhibitors and phosphatase inhibitors (25 mM HEPES (pH 7.0), 20 mM MgCl_2_, and 2 mM DTT). Cells were lysed using ultrasound and then centrifuged at 20,000 × *g* for 20 min at 4 C. For the CETSA melting curve experiment, the cell lysate was diluted in detergent-free buffer, divided into two aliquots, and then treated with or without 50 μM isoimperatorin at room temperature for 30 min. Each sample was divided into 12 small aliquots at 50 μl/tube, heated at different temperatures for 3 min, and then immediately cooled on ice for 3 min. The heated cell extract was centrifuged at 20,000 × *g* at 4 C for 20 min to separate the soluble fraction from the pellet. After centrifugation, the supernatant was analysed by Western blotting with anti-NA antibody. The relative chemiluminescence intensity of each sample at different temperatures was used to generate a temperature-dependent melting curve. The apparent aggregation temperature (T_agg_) value was calculated by nonlinear regression.

### Molecular Docking

The crystal structure of neuraminidase was downloaded from the RSCB Protein Data Bank database (PDB ID: 3TI6). The SDF file of isoimperatorin (PubChem ID: 68081) was downloaded from the PubChem database. The Surflex-dock module of Sybyl-X2.1.1 was used to perform the docking procedure, and the receptor-ligand interaction was displayed through Discovery Studio 2016 software. The semiflexible method was used for docking, and the docking parameters were as follows: expansion factor 1, threshold parameter 0.5, and system default values. Using the total score to evaluate the docking situation, it is generally considered that the score value is proportional to the binding stability of the complex (Li et al., 2013). A score greater than or equal to 6 is considered to indicate good activity, and a score greater than or equal to 9 is considered to indicate very active.

### Statistical Analysis

Statistical analysis was performed using GraphPad Prism 8.0. The experimental results were performed in at least 3 replicates. All data are given as the mean ± SD. Statistical analysis of the results was performed by one-way analysis of variance (ANOVA). *p* values <0.05 were considered statistically significant. *p* values are indicated in the corresponding figure legends.

## Results

### Cytotoxicity of Isoimperatorin in MDCK Cells

**FIGURE 2:**
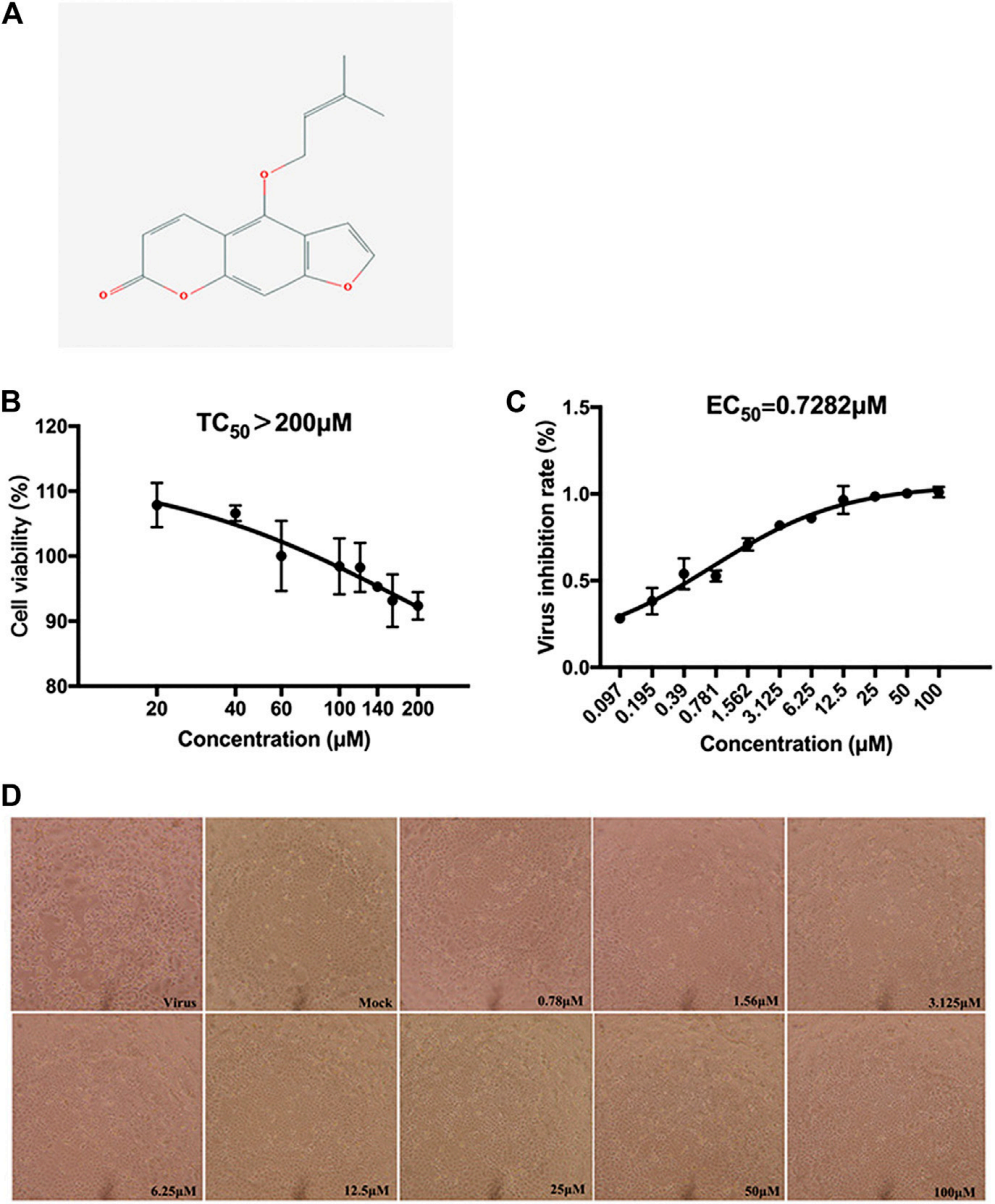
Chemical structure and the inhibition of CPE of isoimperatorin. **(A)** Chemical structure of isoimperatorin. **(B)** Cytotoxicity of isoimperatorin. MDCK cells were treated with a series dilution of isoimperatorin for 48 h and detected by CCK-8 assay. **(C)** MDCK cells were infected with H1N1 A/PR/8/34 influenza virus, and then the antiviral effects of isoimperatorin were detected by CCK-8 assay. **(D)** Virus-induced CPE in different treatments was observed under a microscope. Data are representative of three independent experiments (mean ± SEM). Statistical significance was analysed by one-way ANOVA. ^*#*^ *p* < 0.05, ^*##*^ *p* < 0.01, relative to the mock control group. ^*^ *p* < 0.05, **p < 0.01, relative to the virus group.

The cytotoxicity was accessed by CCK-8 analysis. Isoimperatorin exhibited a low cytotoxic effect on MDCK cells, with a TC_50_ value >200 μM (Figure 2B).

### Anti-Influenza Virus Activity of Isoimperatorin *in vitro*

The CPE reduction assay showed that isoimperatorin could distinctly protect MDCK cells from influenza virus-induced cell cytopathic effects (CPEs) (Figure 2D), with an EC_50_ value of 0.73 μM and SI value > 274.65 (Table 1). The inhibition rates at a concentration of 25 µM against H1N1(A/PR/8/34) reached 98% (Figure 2C).

**TABLE 1:** *In vitro* antiviral activity against H1N1 A/PR/8/34 by isoimperatorin with different medication modes.

Virus	Administration mode	Isoimperatorin (TC_50_ > 200 μM)	
		EC_50_ (μM)	SI (μM)
H1N1 A/PR/8/34	Prophylactic administration	52.08	3.84
	Premixed administration	12.10	16.53
	Therapeutic administration	3.722	53.73

**FIGURE 3:**
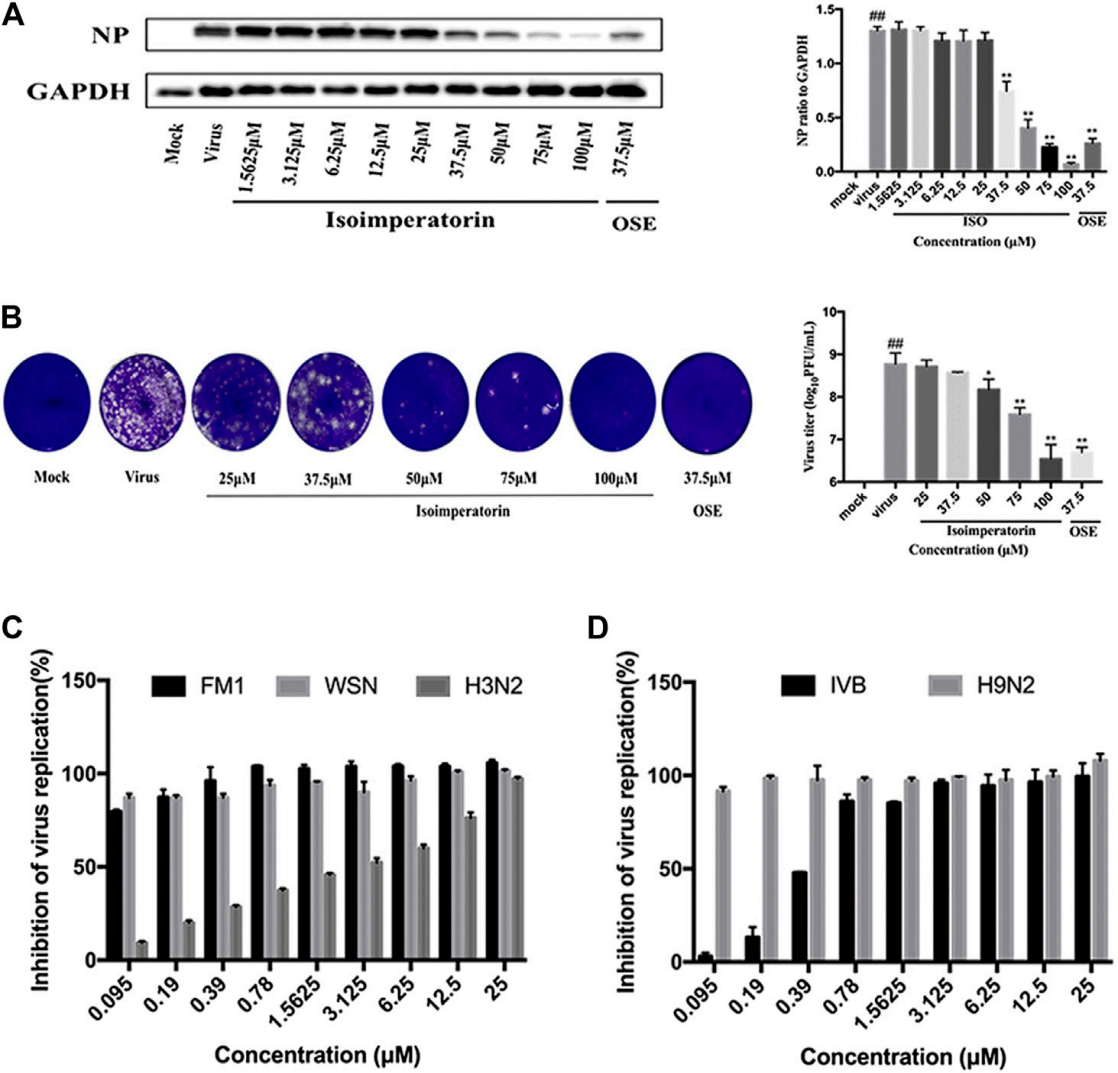
Isoimperatorin inhibits influenza virus infection *in vitro*. **(A)** Isoimperatorin inhibited NP protein expression. Oseltamivir served as a positive control. **(B)** Isoimperatorin reduced the formation of viral plaques. Oseltamivir served as a positive control. **(C)** Antiviral activity of isoimperatorin against different strains of influenza virus. MDCK cells were infected with different strains of influenza virus, and then the antiviral effects of isoimperatorin were detected by CCK-8 assay. Data are representative of three independent experiments (mean ± SEM). Statistical significance was analysed by one-way ANOVA. ^*#*^ *p* < 0.05, ^*##*^ *p* < 0.01, relative to the mock control group. ^*^ *p* < 0.05, ** *p* < 0.01, relative to the virus group.

To further determine the anti-influenza virus efficacy of isoimperatorin, we measured the expression level of viral structural protein NP in the cells and the titre of progeny virus in the cell supernatant 48 h after administration. Oseltamivir (37.5 μM), a commonly used antiviral drug, was regarded as a positive control to confirm the reliability of the assay. As shown in Figure 3A, influenza virus infection resulted in significant expression of nucleoprotein (NP). When the concentration of isoimperatorin reached 37.5 μM, the expression of viral nucleoprotein was significantly inhibited, and it was inhibited in a dose-dependent manner. Plaque reduction assays were used to evaluate the effect of isoimperatorin on the titre of progeny virus in the cell supernatant, and the results were consistent with the expression of viral nucleoprotein (Figure 3B). Furthermore, isoimperatorin showed good antiviral efficacy against different influenza virus subtypes, including influenza A/FM/1/47 (H1N1), A/WSN/33 (H1N1), H3N2, H7N9 and influenza B, as tested by CPE and WST-8 assays (Figures 3C,D). The results showed that cell viability increased as a result of adding isoimperatorin to influenza virus (H3N2 and influenza B)-infected MDCK cells in a dose-dependent manner. However, for H1N1 (A/FM/1/47), A/WSN/33) and H9N2 virus strains, isoimperatorin showed a better inhibitory effect, with at low concentrations (0.39 μM), the inhibition rate of the virus reaching 90%. Taken together, these results indicate that the anti-influenza virus activity of isoimperatorin is not restricted to a single virus strain.

### The Effect of Isoimperatorin on the Virus Replication Cycle

**FIGURE 4:**
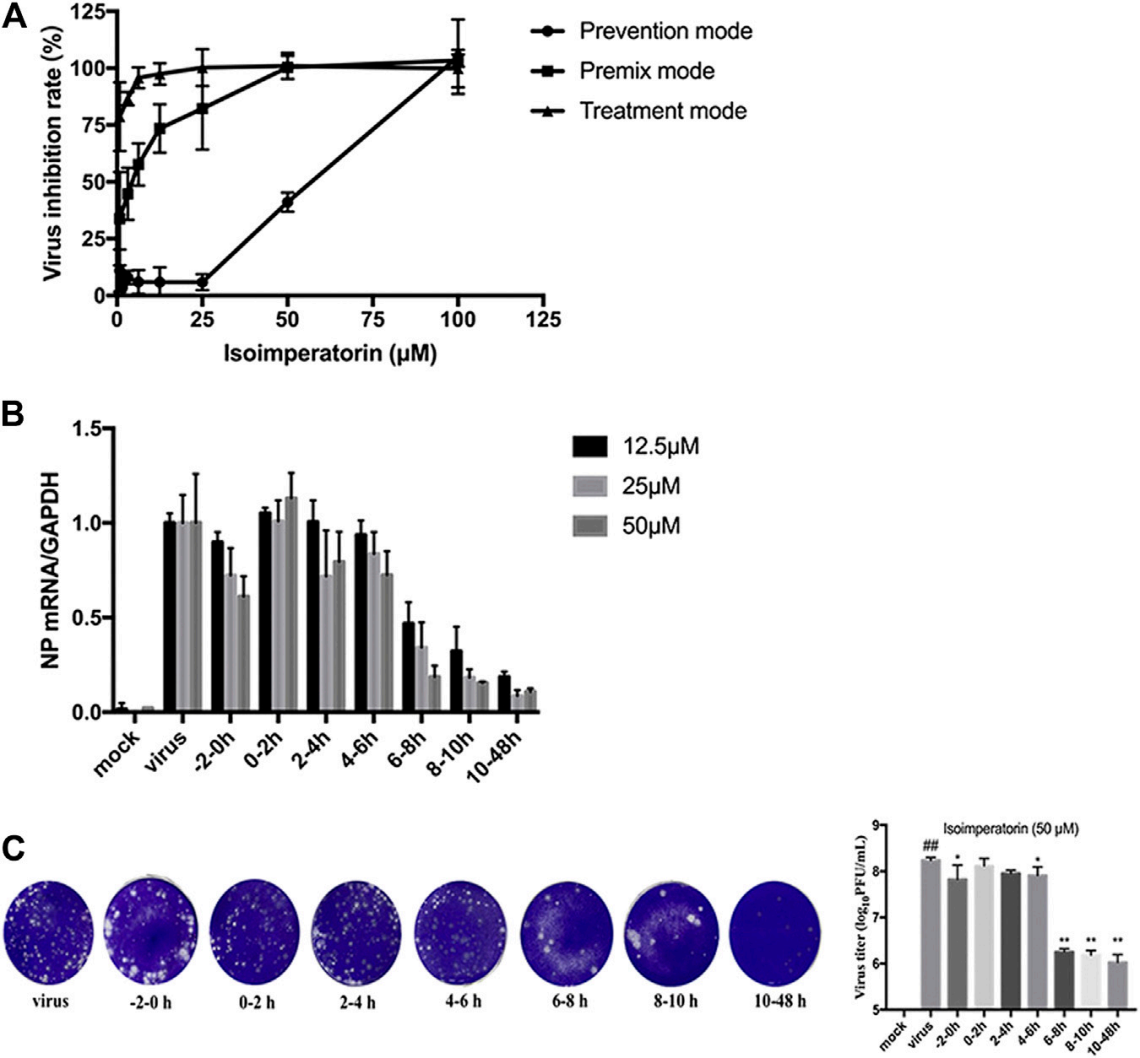
Inhibitory effect of isoimperatorin on the specific steps of the virus lifecycle. **(A)** Different modes of medication. The inhibitory effects of isoimperatorin on H1N1 A/PR/8/34 virus in three medications were detected by CCK-8 assay at 48 h postinfection. **(B,C)** Isoimperatorin inhibits influenza A virus replication at the late stage of the viral lifecycle. Cells were inoculated with 100 μl of 100 TCID_50_ virus. Isoimperatorin was added within the set time, and then the cells were incubated with fresh medium until 48 h after infection. The NP mRNA level was determined by RT-PCR **(B)**, and the virus titre in the supernatant was detected by plaque reduction assay **(C)**. Data are representative of three independent experiments (mean ± SEM). Statistical significance was analysed by one-way ANOVA. ^*#*^ *p* < 0.05, ^*##*^ *p* < 0.01, relative to the mock control group. ^*^ *p* < 0.05, ** *p* < 0.01, relative to the virus group.

To verify the step(s) by which isoimperatorin inhibits the influenza life cycle, we first performed different modes of medication, including prophylactic administration, premixed administration, and therapeutic administration (Figure 1). With different modes of medication, isoimperatorin displayed anti-influenza virus activity (Table 1 and Figure 1). In therapeutic administration and premixed administration, the inhibitory effect of isoimperatorin displayed higher dose dependence (Figure 4A). Notably, in therapeutic administration, isoimperatorin had the highest potency of the three administration modes, with an EC_50_ of 3.722 µM (Figure 4A and Table 2). The selection index (SI) exceeds 53.75, indicating that isoimperatorin may possess a promising safety profile (Zhang et al., 2019).

**TABLE 2:** Inhibition effects of ISO on the A/Puerto Rico/8/34(H1N1) strain. Selectivity index (SI) = EC_50_/TC_50_.

IAV strains	EC_50_ (μM)	TC_50_ (μM)	SI
A/Puerto Rico/8/34(H1N1)	0.73 ± 1.86	>200	>274.65

Then, a time-of-addition experiment of the inhibitory effects of isoimperatorin was carried out. A complete influenza virus life cycle mainly includes viral adsorption, entry, uncoating, biosynthesis and release of progeny virions. It has been reported that approximately 8–10 h is usually required to detect progeny virus after inoculation with the influenza A/PR/8/34 virus (Furuta et al., 2005). We selected seven intervals (−2–, 0–2, 2–4, 4–6, 6–8, 8–10, and 10–48 h) for the inhibition time course by isoimperatorin to determine the NP mRNA levels. As shown in Figure 4B, when isoimperatorin was added at the release stage (6 or more h after infection), the NP mRNA level was significantly reduced, while no inhibitory activity was observed when isoimperatorin was added during the adsorption (0–2 h) stage and the viral replication stage (2–4 or 4–6 h after infection). Compared with the DMSO control, isoimperatorin (50 µM), added at 6–8, 8–10, and 10–48 h intervals, inhibited the mRNA level of the NP gene to 81.4, 84.6, and 89.4%, respectively (Figure 4B). The inhibitory rate was increased in a dose-dependent manner. It is worth noting that when isoimperatorin was added 0–2 h before virus infection, isoimperatorin showed a certain inhibitory effect on the virus, suggesting that it might be related to the regulation of host immunity. In addition, the supernatants were collected for the plaque reduction assay. As we expected, the trend of virus titre in the supernatant was consistent with the expression level of NP mRNA in the cells. In the late stage of virus replication (6–10 h), the virus titre was significantly suppressed (Figure 4C). Taken together, these results suggest that isoimperatorin is effective during the late stage of the viral lifecycle, such as assembly and release.

### Isoimperatorin has No Effect on the Replication of all Three Viral RNA Species.

**FIGURE 5:**
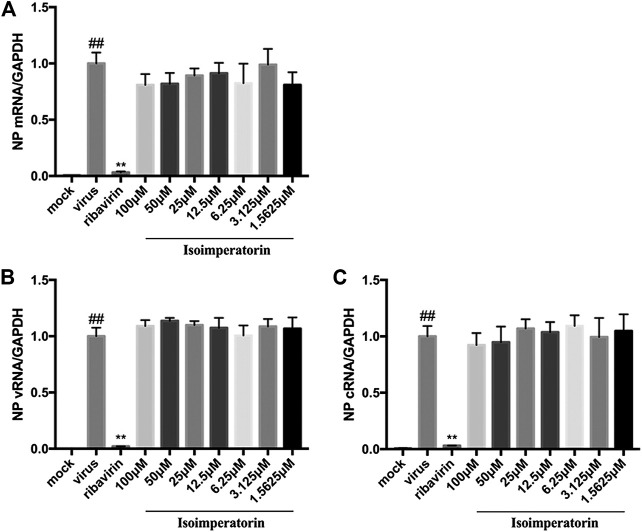
Isoimperatorin has no effect on the replication of all three viral RNA species. **(A)** mRNA expression level. **(B)** vRNA expression level. **(C)** cRNA expression level. MDCK cells were infected with H1N1(A/PR/8/34) virus at 100 TCID50 and then treated with DMSO control, ribavirin control (35.5 μM), or various concentrations of isoimperatorin at 2 hpi. Total RNA was collected at 6 hpi, and viral RNA levels were quantified by real-time RT-PCR. The RNA expression level of the DMSO control group (0 μM) was set as 100%. Data are representative of three independent experiments (mean ± SEM). Statistical significance was analysed by one-way ANOVA. ^*#*^ *P* < 0.05, ^*##*^ *P* < 0.01, relative to the mock control group. ^*^ *P* < 0.05, ** *P* < 0.01, relative to the virus group.

To investigate whether isoimperatorin directly inhibits influenza viral RNA synthesis, MDCK cells were infected with H1N1 A/PR/8/34 virus for 2 h and then treated with a series of gradient concentrations of isoimperatorin or ribavirin (35.5 μM) for 6 h. Total RNA from the cells was extracted for quantitative analysis of mRNA, vRNA, and cRNA. The levels of all three RNA species were not inhibited by isoimperatorin but were significantly decreased with the addition of ribavirin (35.5 μM), a known inhibitor of influenza virus RNA synthesis (Figure 5). These results suggest that isoimperatorin is not effective in viral RNA synthesis.

### Isoimperatorin Blocks Virion Release by Targeting NA

**FIGURE 6:**
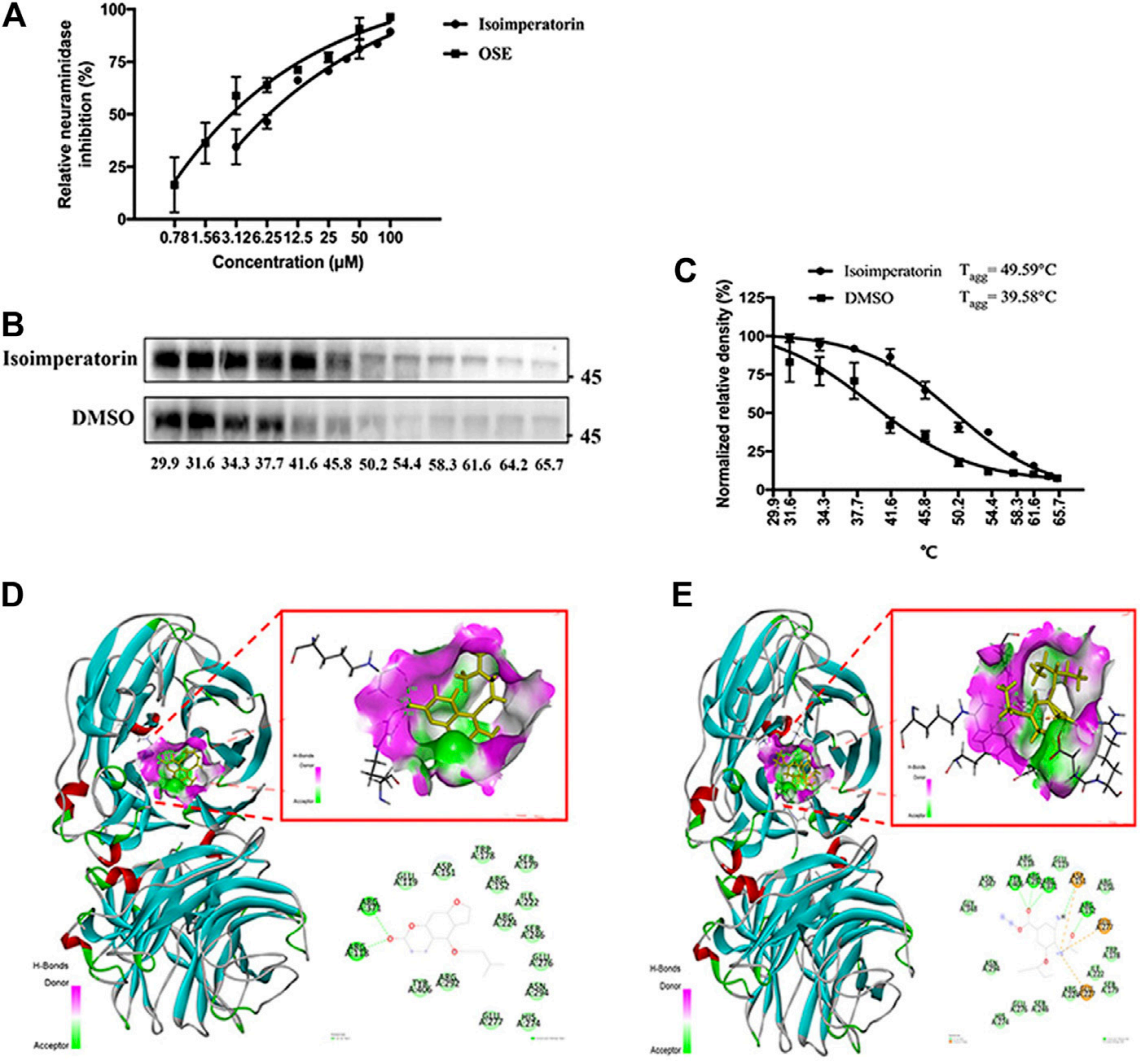
Isoimperatorin inhibits influenza by targeting NA. **(A)** The inhibitory effect of isoimperatorin on the NA of H1N1 A/PR/8/34 influenza virus was detected by ELISA. Oseltamivir served as a positive control in the NA inhibition test. **(B)** Western blotting showing the H1N1(A/PR/8/34) NA CETSA binding assay in the presence or absence of 50 μM isoimperatorin at different temperatures. **(C)** The NA band intensities in the CETSA were quantified. **(D–E)** The predicted binding models (3D and 2D) between neuraminidase and compounds. Data are representative of three independent experiments (mean ± SEM).

Neuraminidase plays an important role in the life cycle of influenza viruses, which can catalyse the hydrolysis of sialic acid and assist mature influenza viruses in escaping from host cells and infecting new cells (Byrd-Leotis et al., 2017). To determine whether isoimperatorin can inhibit neuraminidase, we conducted an NA inhibition assay (Chintakrindi et al., 2020). As a result, isoimperatorin significantly inhibited NA activity in a dose-dependent manner (Figure 6A), with an IC_50_ value of 7.17 ± 1.99 μM (Table 3). Oseltamivir, a recognized NA inhibitor, served as a positive control with an IC_50_ value of 1.97 ± 3.10 μM (Table 3). To further investigate the binding ability of intracellular isoimperatorin and NA target proteins, we performed a CETSA (Martinez Molina et al., 2013). T_agg_ values of NA in MDCK cells were measured in the absence or presence of isoimperatorin at temperatures ranging from 29.9 to 65.7°C (Figure 6B). As shown in Figure 5C, isoimperatorin (50 μM) significantly enhanced the thermal stability of NA, increasing the T_agg_ of the NA protein from 48.58 to 49.59°C (Figure 6C).

**TABLE 3:** Inhibition effects of compounds on neuraminidase.

Compounds	EC_50_ (μM)
Isoimperatorin	7.17 ± 1.99
Oseltamivir	1.97 ± 3.10

To verify the inhibition mechanism of isoimperatorin on NA, we carried out molecular docking to simulate the interaction between isoimperatorin and NA protein amino acid residues by using Sybyl-X2.1.1. Here, oseltamivir was chosen as the positive control to assess the ability of isoimperatorin to bind to NA. The total score was used to evaluate the binding of the protein-ligand complex. A score of >6 is considered to indicate better activity, and a score of ≥9 is considered to indicate very good activity (Golbraikh and Tropsha, 2002). The total score between isoimperatorin and NA was 6.959, while oseltamivir reached 10.4036. The 3D and 2D docking models of compounds and NA are shown in Figures 5D,E. There were two conventional hydrogen bonds between isoimperatorin and NA, indicating that the H-bond of this portion of the binding region played an important role in the stability of the complex. As a positive control, oseltamivir exhibited better binding activity for NA, with five conventional hydrogen bonds, one carbon hydrogen bond, and one attractive charge, which was consistent with the NA inhibition assay (Table 4). All of these data indicated that isoimperatorin targets NA and potently inhibits NA activity.

**TABLE 4:** The total scores and residues for the chemical bonds between compounds and neuraminidase.

Compounds	Total score	Conventional hydrogen bond residues	Carbon hydrogen bond residues	Attractive charge
Isoimperatorin	6.959	ARG: 371, ARG: 118	-	-
Oseltamivir	10.4036	ARG: 152, ARG: 371, ARG: 118, ARG: 292, GLU: 119	TYR: 406	ASP: 151, GLU: 277, GLU: 227

## Discussion

There are two glycoproteins on the surface envelope of influenza virus, hemagglutinin and neuraminidase. Haemagglutinin is responsible for virus attachment to cell surface receptors, which are terminal sialic acid residues usually linked to galactose. Neuraminidase destroys the virus receptor by cleaving the glycosidic bond between hemagglutinin and sialic acid, thereby promoting the release of progeny viruses from infected host cells. Therefore, scientists recognize that neuraminidase is a key target in the viral replication cycle. At present, zanamivir and oseltamivir are promising long-acting neuraminidase inhibitors for the prevention and treatment of influenza (De Clercq and Li, 2016). However, the emergence of drug-resistant strains makes the development of new neuraminidase inhibitors more urgent. It is worth noting that some natural products have also been proven to have NA inhibitory activity. Hence, it is of great significance to discover new neuraminidase inhibitors (NAIs) from natural products.

Coumarin compounds are an important class of natural products that have a variety of pharmacological effects, including antioxidant (Wahy et al., 2017), carbonic anhydrase inhibition (Ferraroni et al., 2016; De Luca et al., 2018), antibacterial (Souza et al., 2005; Nagamallu et al., 2016), antifungal (Tiwari et al., 2017), neuroprotection (De Souza et al., 2016; Najafi et al., 2019), anticonvulsant (Abd-Allah et al., 2020), antidiabetic (Salar et al., 2016; Taha et al., 2018; Dhameja and Gupta, 2019; Pillai et al., 2019), anticoagulant (Nieto et al., 2019), anti-inflammatory (Liu et al., 2019), antitumour (Dandriyal et al., 2016) and antiviral activities (Khomenko et al., 2017; Annunziata et al., 2020). Studies have found that coumarin compounds have inhibitory effects on a variety of viruses, such as inhibiting HIV reverse transcription (Liu et al., 2019), anti-measles virus (Barnard et al., 2002), HSV (Shokoohinia et al., 2014), hepatitis B virus (Huang et al., 2019) and influenza virus (Wang et al., 2017). For example, eleutheroside B1 showed a wide spectrum of anti-human influenza virus effects by inhibiting viral NP and anti-inflammatory activity (Wang et al., 2017). One of the bis-coumarinyl-bis-triazolothiadiazinyl ethane derivatives, compound 83, exhibited good antiviral activity against influenza A virus, with EC_50_ values of 20–72 μM (Pavurala et al., 2018). Bizzarri et al. exploited the regioselective oxidation of coumarin derivatives with 2-iodoxybenzoic acid (IBX) and found that pyrogallol derivatives 88 (IC_50_ = 69.9 μg/ml) and 89 (IC_50_ = 47.9 μg/ml) were more active than catechol derivatives 84 (IC_50_ = 106.5 μg/ml) and 85 (IC_50_ = 91.5 μg/ml) (Bizzarri et al., 2017). Previous studies reported that isoimperatorin exhibits strong anti-influenza virus (H1N1, H9N2) activities *in vitro* (Lee et al., 2020), with EC_50_ values (7.67 ± 0.93 μM and 6.72 ± 0.51 μM, respectively) comparable to that of ribavirin. However, the antiviral mechanism of isoimperatorin is not clear. In this study, the potent anti-influenza virus mechanism of isoimperatorin was reported for the first time. Our results indicated that isoimperatorin showed strong activity against influenza A/PR/8/34 virus and that the degree of antiviral activity varied in a dose-dependent manner. A CPE reduction experiment showed that isoimperatorin could completely protect MDCK cells from H1N1 influenza virus at a concentration of 25 μM, and its half effective concentration was 0.73 μM. In the concentration range of 37.5–100 μM, isoimperatorin effectively downregulated the expression of influenza virus NP protein (*p* < 0.01) and achieved similar efficacy to oseltamivir (37.5 μM) at a concentration of 75 μM. In addition, isoimperatorin significantly inhibited the generation of progeny virus in the cell supernatant at a concentration of 50–100 μM (*p* < 0.05), and the virus titre at a concentration of 100 μM was close to that of oseltamivir (37.5 μM). Additionally, isoimperatorin was sensitive to the different IAV strains with low cytotoxicity, including A/FM/1/47(H1N1), A/WSN/33 (H1N1, S31N, amantadine resistant), and A/Hong Kong/498/97(H3N2), influenza B/Lee/1940(IVB)and A/Chicken/Guangdong/1996(H9N2) virus strains. Three administration mode assays demonstrated that isoimperatorin might inhibit virus replication by affecting the middle and late stages of virus replication. Furthermore, the time of addition assay results suggested that isoimperatorin effectively suppressed virus replication by 84.6% when added at 8–10 h intervals. As expected, isoimperatorin showed no effect on the replication of all three viral RNA species (mRNA, vRNA, cRNA) (*p* > 0.05), which indicates that isoimperatorin does not directly inhibit influenza viral RNA synthesis. Neuraminidase inhibition experiments showed that isoimperatorin inhibited NA activity in a dose-dependent manner, with an IC_50_ of 7.17 μM < 1.97 μM (oseltamivir). CETSA further confirmed that isoimperatorin could bind to NA, raising the Tagg value from 48.58 to 49.59°C. The docking study indicated that isoimperatorin could bind to the NA protein through H-bonds. In this study, we proved that isoimperatorin could be a potential NAI that can reduce the release of virus by inhibiting NA activity in a dose-dependent manner and has a strong interaction with NA mainly through conventional hydrogen bonds and van der Waals forces.

In summary, our study demonstrates that isoimperatorin, a natural coumarin compound derived from the traditional Chinese herbal medicines *Angelica dahurica*, *Rhizoma et Radix Notopterygii* and *Radix Peucedani*, could effectively inhibit influenza virus replication by blocking the NA protein, suggesting that isoimperatorin may be exploited as a potential antiviral compound in the pharmaceutical industry.

## Data Availability

The original contributions presented in the study are included in the article/Supplementary Material, further inquiries can be directed to the corresponding authors.
